# The International Congress of Medicine, Lisbon, 1906

**Published:** 1905-02-11

**Authors:** 


### THE INTERNATIONAL CONGRESS OF MEDICINE, LISBON, 1906.

A meeting of the National Committee for Great Britain and Ireland of the Fifteenth International Congress of Medicine, to be held at Lisbon from April 19th to 26th, 1906, took place in the rooms of the Medical Society of London on February 3rd, 1905. There were present amongst others Dr. F. W. Pavy, F.R.S., president of the National Committee, Sir W. S. Church, Bart., K.C.B., Sir Dyce Duckworth, M.D., Dr. Ferrier, F.R.S., Dr. Gordon Dill, Dr. Radcliffe Crocker, Dr. Boyd Joll, Mr. W. H. H. Jessop; and the honorary secretaries, Mr. D'Arcy Power and Dr. Clive Riviere.

It was announced that the following gentlemen had undertaken to act as *rapporteurs* at the forthcoming Congress, as it was the wish of the Lisbon Committee to make the *rapports* a special feature of the meeting. The subject in each case had been chosen by the Lisbon Committee. Dr. Stanley B. Atkinson, barrister-at-law and Hon. Secretary of the Medico-Legal Society, "Spontaneous and Criminal Abortion in its Medico-Legal Aspect"; Dr. Chalmers, medical officer of health for the City of Glasgow, "The Administrative Control and Technique of Public Disinfection"; Mr. Treacher Collins, F.R.C.S.Eng., "Ocular Tuberculosis"; Professor Ferrier, F.R.S., "The Nature and Pathological Physiology of Tabes"; Mr. Goadby, lecturer on bacteriology at the National Dental Hospital, London, "The Treatment of Pyorrhœa Alveolaris"; Dr. Gulland, "The Classification, Origin, and probable rôle of Leucocytes, Mastzellen and Plasma Cells"; Dr. Mott, F.R.S., "Cerebral Lesions in Psychoses of Toxic Origin"; Mr. D'Arcy Power, F.R.C.S.Eng., "Recent Advances in the Surgery of the Arteries and Veins"; Dr. Still, "The Spastic Affections of Childhood, their Classification and Pathogeny"; Dr. Sutherland, deputy commissioner in lunacy for Scotland, "The Prophylaxis and Treatment of Habitual Criminals"; Mr. C. E. West, F.R C.S.Eng., "A Study of the Eleptogenous Action of Foreign Bodies in the Ear and of Postnasal Growths." Sir Patrick Manson, K.C.M.G., had undertaken to open a conference upon some subject connected with tropical medicine.

A letter was read from Messrs. Thomas Cook and Son offering to provide a first-class passenger ship to convey visitors to and from Lisbon, calling at Corunna, Vigo, and Oporto, and acting as a floating hotel during the Congress week, the whole trip to London and back taking 18 days, and at an inclusive charge. The suggestion was approved by the committee, but Messrs. Cook were desired to ascertain the exact position in the Tagus at which anchorage would be allowed, that inquiries might be made locally as to its suitability, both from the point of view of the hygienic surroundings and of the distance from the seat of the Congress meetings.

Letters were read from Dr. Ogilvie Will, of Aberdeen, and Dr. J. F. Sutherland, of Edinburgh, giving the results of inquiries which had been made in regard to the housing of those who did not go by sea or who would not remain on the boat whilst at Lisbon. It appeared from these inquiries that rooms would be very difficult to obtain, but there were still some to be had at a moderate rate at Cintra, which is about 40 minutes by train from Lisbon. The proprietor of Netto's Hotel says that at present he has 16 bedrooms, most of which can be used by two persons. It appears imperative, therefore, that those who intend to go to the Congress and do not wish to depend on the efforts of the local committee to obtain lodgings should at once take the opportunity of securing rooms. Delay is likely to lead to the same disastrous consequences as at Madrid.

